# Parallel evolution, atavism, and extensive introgression explain the radiation of *Epimedium* sect. *Diphyllon* (Berberidaceae) in southern East Asia

**DOI:** 10.3389/fpls.2023.1234148

**Published:** 2023-10-17

**Authors:** Cheng Zhang, Ran Meng, Ying Meng, Bao-Lin Guo, Quan-Ru Liu, Ze-Long Nie

**Affiliations:** ^1^ Key Laboratory of Biodiversity Science and Ecological Engineering of Ministry of Education, College of Life Sciences, Beijing Normal University, Beijing, China; ^2^ College of Biological Resources and Environmental Sciences, Jishou University, Jishou, Hunan, China; ^3^ Institute of Medicinal Plant Development, Chinese Academy of Medical Sciences & Peking Union Medical College, Beijing, China

**Keywords:** East Asia, evolutionary radiation, atavism, introgression, parallel evolution, *Epimedium*, *Epimedium* sect. *Diphyllon*

## Abstract

East Asia is the richest region of plant biodiversity in the northern temperate zone, and its radiation provides key insights for understanding rapid speciation, including evolutionary patterns and processes. However, it is challenging to investigate the recent evolutionary radiation among plants because of the lack of genetic divergence, phenotypic convergence, and interspecific gene flow. *Epimedium* sect. *Diphyllon* is a rarely studied plant lineage endemic to East Asia, especially highly diversified in its southern part. In this study, we report a robust phylogenomic analysis based on genotyping-by-sequencing data of this lineage. The results revealed a clear biogeographic pattern for *Epimedium* sect. *Diphyllon* with recognition into two major clades corresponding to the Sino–Himalayan and Sino–Japanese subkingdoms of East Asian Flora and rapid diversification of the extant species dated to the Pleistocene. Evolutionary radiation of *Epimedium* sect. *Diphyllon* is characterized by recent and predominant parallel evolution and atavism between the two subkingdom regions, with extensive reticulating hybridization within each region during the course of diversification in southern East Asia. A parallel-atavism-introgression hypothesis is referred to in explaining the radiation of plant diversity in southern East Asia, which represents a potential model for the rapid diversification of plants under global climate cooling in the late Tertiary. Our study advances our understanding of the evolutionary processes of plant radiation in East Asia as well as in other biodiversity hotspot regions.

## 1 Introduction

East Asia (EA) is the richest region in the Northern Hemisphere in terms of the biogeographic diversification of flowering plants (Takhtajan, 1986; Wu and Wu, 1998) and has been considered a massive sanctuary for Tertiary relict flora and associated forest plants during the climatic fluctuations over the Neogene (Tiffney, 1985; Liu, 1988; Wen, 1999). EA has been a natural floristic region since Grisebach first discussed it in 1872 (Takhtajan, 1986; Wu, 1988), well known for its abundant endemic elements from more than 31 families and 600 genera of flowering plants (Good, 1964; Takhtajan, 1969; Takhtajan, 1986; Wu and Wu, 1998; Qian, 2002). The high species richness in EA can be attributed to secondary diversification due to habitat heterogeneity and a lower extinction rate in the Neogene, whereas the lower species diversity in North America and Europe is often explained by the hypothesis that more severe extinctions occurred as a consequence of global cooling in the late Eocene to Oligocene (Xiang et al., 2004; Wen et al., 2010). It has also been suggested as the ancestral area for most EA–North American disjunct lineages (Wen, 1999; Milne and Abbott, 2002). Biogeographic studies on flowering plants in the Northern Hemisphere have largely focused on the classic EA and North American disjunctions; however, few studies have explored the morphological and evolutionary patterns and mechanisms to explain the radiation and high diversity of EA flora (Wen, 1999; Donoghue et al., 2001).

Evolutionary radiation, a widely recognized mode of rapid species diversification, is a fascinating phenomenon for biologists. Recently, the mechanisms underlying evolutionary radiation have been extensively studied, and several lineages of organisms have been characterized by rapid evolutionary radiation, such as Darwin’s finches (Grant and Grant, 2002; Lamichhaney et al., 2015), African lake cichlids (Seehausen, 2006; Wagner et al., 2012) and representatives of Andean flora (Pennington et al., 2010; Hughes and Atchison, 2015). Introgression and hybridization are common in plant evolution, especially in radiation lineages (Ellstrand and Schierenbeck, 2000; Linder and Rieseberg, 2004; Vriesendorp and Bakker, 2005; Soltis and Soltis, 2009; Harrison and Larson, 2014; Vargas et al., 2017). Hybridization and introgression often lead to chromosomal rearrangements, whole-genome duplication, and differential gene expression; some of which play important roles in plant speciation and adaptive radiation (Linder and Rieseberg, 2004; Seehausen, 2004; Baack and Rieseberg, 2007; Alix et al., 2017). Interspecific hybridization has been estimated to have occurred in at least 25% of flowering plant species (Mallet, 2005; Mallet, 2007).

**Figure 1:**
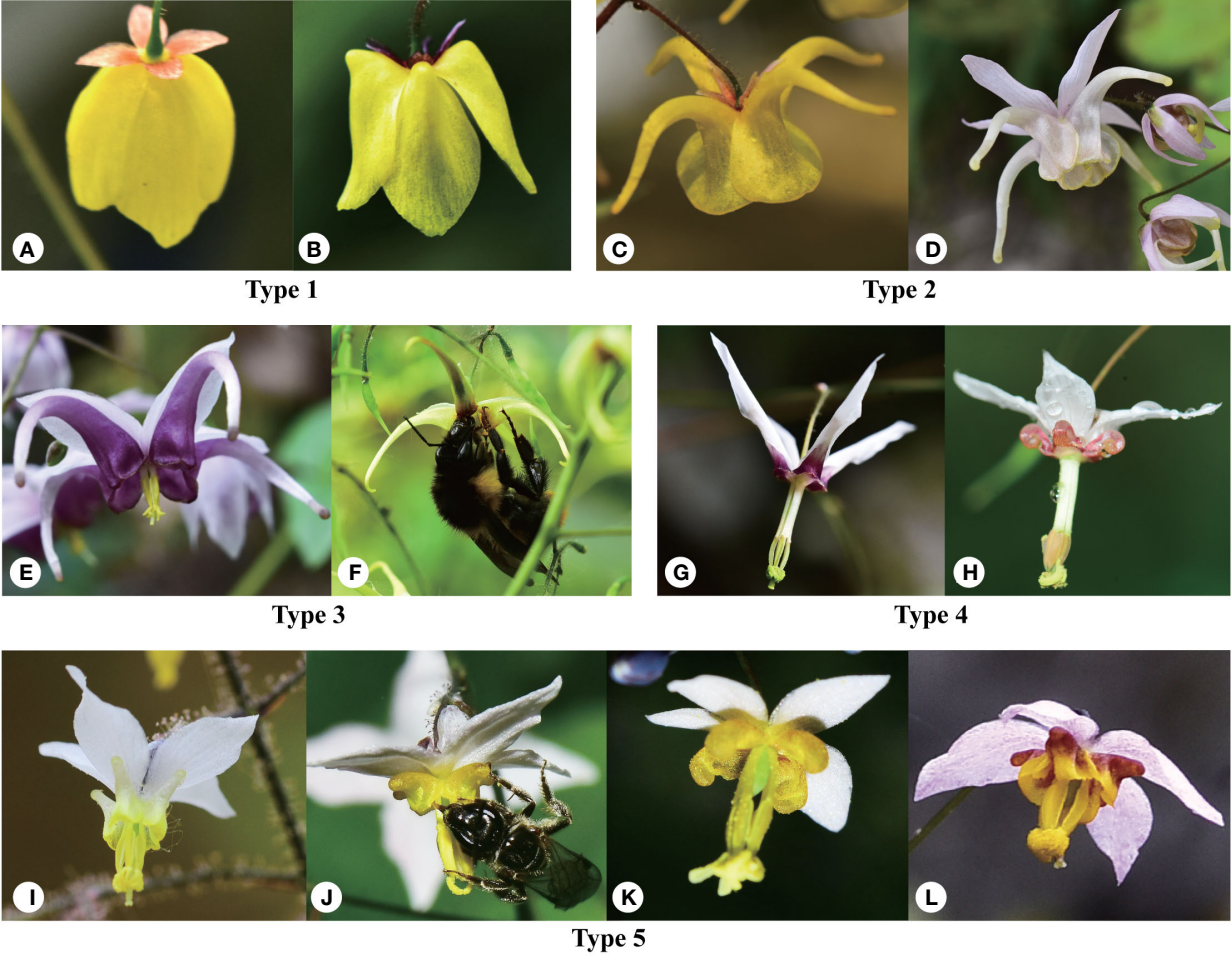
Morphological diversity of *Epimedium* sect. *Diphyllon* showing the five types of flowers and pollinators. Type 1: lamina flat, obovate, with a slight median nectariferous furrow but no spur or a saccate spur. **(A)** *E*. *campanulatum*, **(B)** *E*. *ecalcaratum*. Type 2: lamina rounded and conspicuous but expanded outwards at base into an elongated spur usually longer than the inner sepal. **(C)** *E. davidii*, **(D)** *E. hunanense*. Type 3: lamina reduced to a rim around the mouth of the elongated basally-swollen spur which is longer than the inner sepal. **(E)** *E. aciminatum*, **(F)** *E. jingchengshanense*. Type 4: lamina reduced to a rim around the mouth of the slipper-like cylindric spur, which is shorter than inner sepal. **(G)** *E. fargesii*, **(H)** *E. dolichostemon*. Type 5: lamina rand spur alike reduced, forming a minute pouch. **(I)** *E*. *brevicornu*, **(J)** *E. stellulatum*, **(K)** *E. myrianthum, and* **(L)** *E. truncatum*.

*Epimedium* sect. *Diphyllon* from Berberidaceae is an excellent model for studying species radiation with possible network evolution in southern EA. First, *Epimedium* sect. *Diphyllon* is the largest section within the morphologically unique genus of *Epimedium* characterized by spider-like flowers (**Figure 1**). This section encompasses over 80% of all *Epimedium* species, which is primarily distributed within southern EA from southwestern, central to southeastern China. The section has two main distribution centers of Hengduan Mountain region and south-central China, located on each side of the boundary between the Sino–Japanese Forest and the Sino–Himalayan Forest subkingdoms in the East Asian Flora (Wu and Wu, 1998), providing a good chance to test the possible link between floristic division and taxa phylogenetic relationships.

Furthermore, species delimitation and morphological variations in the speciose *Epimedium* sect. *Diphyllon* are extremely complicated owing to their diverse morphological traits, which vary widely in petal type, form, relative size of inner sepals and petals, and flower dimensions (Ying, 2002; Zhang et al., 2007; De Smet et al., 2012; Zhang et al., 2014b). Both nuclear and chloroplast data suggest that *Epimedium* sect. *Diphyllon* is a monophyletic lineage with a recent evolutionary history (Sun et al., 2005; Zhang et al., 2007; De Smet et al., 2012; Zhang et al., 2014b; Zhang et al., 2021). Traditionally, the section is classified into four series (*Campanulatae, Davidianae, Dolichocerae*, and *Brachycerae*) based on flower morphology (Stearn, 2002). However, phylogenetic studies have not confirmed the classification and phylogeny within the *Epimedium* sect. *Diphyllon* based on pollen types, flavonoid types, karyotypes, and molecular markers (Guo and Xiao, 1999; Sun et al., 2005; Sheng and Chen, 2007; Zhang et al., 2007; Guo et al., 2008; Zhang et al., 2008; Sheng et al., 2010; De Smet et al., 2012; Zhang et al., 2018). The backbone and species relationships in this section remain unclear, as evidenced by species from the same series that are not clustered into one clade (Zhang et al., 2014b). Despite recent phylogenetic analysis from the plastid genome sequences provided much better phylogenetic resolution within *Epimedium* sect. *Diphyllon*, the results could not provide a clear route for morphological evolution in *Epimedium* sect. *Diphyllon* (Guo et al., 2021). Further investigation of morphological diversity and evolution in EA is necessary, building on robust phylogenetic relationships from nuclear genomic data.

Third, possible hybridization or introgression among species has been frequently suggested for the *Epimedium* sect. *Diphyllon.* An outcrossing breeding system with strong self-incompatibility and high cross-compatibility between infraspecific and interspecific individuals is prevalent in *Epimedium* (Suzuki, 1984; Sheng et al., 2011). Observations of garden experiences have also indicated that all *Epimedium* species might be interfertile when brought together (Stearn, 2002; Sheng et al., 2011). *Epimedium* species rarely overlap in the different areas they occupy, and they seemingly evolved in geographical isolation although sympatric species are also common and may have produced several hybrid swarms. For example, the meeting of *E. acuminatum* and *E. fangii* on Mt. Omei resulted in the creation of a hybrid swarm: *E.*×*omeiense* (Stearn, 1995). The interspecies similarity of *Epimedium* is close to or even higher than the intraspecies similarity (Guo et al., 2021). Frequent gene flow among sympatric populations due to pollen or seed dispersal might be one of the reasons for the low interspecific differentiation among *Epimedium* species (Xu et al., 2007). We hypothesized that introgression and hybridization played an important role in evolution and triggered rapid species diversification and diverse phenotypic traits in our studied group.

Genome-wide introgression signals are difficult to detect, especially in older interspecific hybridization events or rapidly diverged lineages (Soltis and Soltis, 2009). Robust phylogenetic inferences are required to understand mechanisms underlying species evolution. In plants, unfortunately, most groups that rapidly speciated exhibit a low resolution of interspecific relationships with a wide occurrence of polytomy and conflicting phylogenies due to a lack of informative loci, frequently accompanied by incomplete lineage sorting (ILS) and/or hybridization including introgression in the early reticulate history. Thus, more robust data and analyses are required to explore hybridization events and distinguish ILS from hybridization and introgression (Vargas et al., 2017).

Studying the evolutionary history of *Epimedium* sect. *Diphyllon* proves to be challenging because of its recent and rapid radiation, potential phenotypic convergence, and limited informative loci from nrDNA, cpDNA, and AFLP data (Sun et al., 2005; Zhang et al., 2007; De Smet et al., 2012; Zhang et al., 2014b; Zhang et al., 2021), which resulted in polytomies of the phylogenetic tree, making researches unable to generate strong phylogenetic hypotheses. Here, we applied the genotyping-by-sequencing (GBS) strategy to collect the genomic sequences of *Epimedium* sect. *Diphyllon* with the integration of morphological evidence; then, we employed concatenated, coalescent-based, biogeographic, and phylogenetic network methods to determine the underlying mechanism of phenotypic evolution and recent rapid radiation in the evolutionary history of *Epimedium* sect. *Diphyllon* in southern EA. Our study sheds light on the processes of plant radiation patterns and reveals possible mechanisms underlying the high level of plant diversity in this region.

## 2 Materials and methods

### 2.1 Taxa sampling

We sampled 57 accessions for GBS sequencing, including 43 species and 2 putative new species representing the taxa from all four series of *Epimedium* sect. *Diphyllon* (*Campanulatae*: 2 species, *Davidianae*: 14 species, *Dolichocerae*: 14 species, and *Brachycerae*: 14 species), and one species from sect. *Maceceras* (*E. koreanum*) was used as an outgroup (**Supplementary Table S1**). Voucher specimens were deposited in the Herbarium of Beijing Normal University. Taxonomic systems and species identification were largely based on Stearn (2002) except for the seven recently published species that were recognized following the original literature (Guo et al., 2007; Zhang and Li, 2009; Zhang et al., 2014a; Zhang et al., 2015; Zhang et al., 2016).

### 2.2 DNA extraction and GBS

Total genomic DNA was extracted from approximately 20 mg of fresh leaf tissue from each plant using a modified CTAB protocol (Doyle and Doyle, 1987; Cullings, 1992). DNA concentrations were quantified using a Qubit 2.0 fluorometer (Thermo Fisher Scientific Inc., California, USA) with a dsDNA Broad-Range Assay Kit.

The GBS library was prepared from DNA samples using the PstI-HF restriction enzyme according to previously published protocols (Elshire et al., 2011; Escudero et al., 2014), with some modifications. For each sample, 500 ng of genomic DNA was combined with 0.6 pmol of a sample-specific barcode adapter and 0.6 pmol of a common adapter. Each sample was digested with four units of PstI-HF (New England Biolabs Inc., Massachusetts, USA) at 37°C overnight. The adapters were ligated using 400 units of T4 DNA Ligase (New England Biolabs Inc., Massachusetts, USA) at room temperature for 4 h. We combined 50 ng of each sample, and the pool was purified using Agencourt AMPure XP (Beckman Coulter, Inc., Indiana, USA). The DNA fragments were amplified for 15 PCR cycles starting from 35 ng of DNA using NEB 2x Taq MasterMix (New England Biolabs Inc., Massachusetts, USA). The PCR product was purified using different volume ratios of AMPure XP beads to sample, and fragment size distributions were assessed using a 2100 Bioanalyzer (Agilent Technologies, Inc., California, USA). The library with the optimal profile and concentration (AMPure to sample ratio: 0.8:1, concentration: 1.87 ng/μl, average size: 595 bp) was submitted to Majorbio Bio-Pharm Technology (Shanghai, China) for 100 bp HiSeq 2000 Illumina sequencing. Quality control of the sequencing results was performed using FastQC 0.11.3 (Andrews, 2010).

The assembly of GBS loci was performed using the ipyrad 0.9.30 pipeline (Eaton and Overcast, 2020), which allows for indel variation (commonly found in phylogenetic datasets) and is optimized for downstream phylogenetic analyses. We conducted a *de novo* assembly of the GBS data. To compare and verify the *de novo* assembly results, a second assembly was performed based on the reference genome by mapping the full-length transcriptome sequence of *E. pseudowushanense* (Pan et al., 2017). The following parameters were applied to assemble the complete loci and single nucleotide polymorphism (SNP) sequences using ipyrad: a maximum of 4 low-quality base calls, a minimum depth of 6 for statistical and majority rule base calling, a maximum clustering depth of 1,000 within samples, 0 mismatches allowed in barcodes, a 50 base-pair minimum length of reads after trimming, and a maximum of 10 SNPs per locus. A minimum sample coverage per locus was set to 43 (ca. 75% of the total) considering the balance between locus coverage and data resolution, which is much higher than those in Fernández-Mazuecos et al. (2020). Finally, we obtained four GBS datasets for downstream analysis: complete *de novo* sequences (Denovo), *de novo* SNPs (Denovo_SNP), complete reference-based sequences (Ref), and reference-based SNPs (Ref_SNP).

### 2.3 Phylogenetic analyses

The gene trees of the four GBS datasets were analyzed using the maximum likelihood (ML) method, as implemented in both RAxML 8.2.10 (Stamatakis, 2014) and IQ-TREE v1.6.10 (Hoang et al., 2018). All datasets were analyzed without partitioning. A rapid bootstrap search (100 replicates), followed by a thorough ML search (-m GTRGAMMA), was implemented for the RAxML approach. The IQ-TREE analysis employed an ML search with the best-fit substitution model automatically selected, and branch support was assessed using an ultrafast bootstrap (Minh et al., 2013). The datasets were also inferred using Bayesian inference (BI), as implemented in ExaBayes 1.4.1 (Aberer et al., 2014) with the GTRGAMMA substitution model.

Species trees were inferred using the SVD quartets method (Chifman and Kubatko, 2014; Chifman and Kubatko, 2015). It is well known that gene trees, especially the rapid radiation group that frequently accompanies ILS, may result in obscure phylogenetic topologies. The SVD quartets method is run under a multispecies coalescent approach and accounts for the ILS. Analyses were performed in PAUP* 4 under multispecies coalescent conditions to evaluate all possible quartets. One hundred bootstrap replicates were conducted and the results were summarized in a 50% consensus tree.

### 2.4 Morphological data and analyses

Two morphometric datasets (vegetative and reproductive traits) of *Epimedium* sect. *Diphyllon* were assembled for morphological analysis (**Supplementary Table S2**). The characteristics of leaves covering 42 species and an unknown species representative of the four series were appraised based on dried, pressed specimens obtained from our field collection and herbarium. The selected characters included 11 variables, and the final morphometric data set included 737 samples (**Supplementary Table S3**). The characteristics of the flowers covering 41 species and one unknown species representative of the four series were assessed based on living plants obtained from our field collection; the selected characters included 11 variables, and the final morphometric dataset included 699 individuals (**Supplementary Table S4**).

To assess the reproductive and vegetative morphological affinities of the four series of *Epimedium* sect. *Diphyllon* discriminant function analysis (DFA) was performed to identify the main characteristics contributing to the morphological differentiation of the taxa. We used the basic package MASS (Venables and Ripley, 2002) of R v3.2.5 (R Development Core Team, 2016) for the DFA. The R package ggord (Beck, 2017) was used to represent the first two axes of the DFA (**Supplementary Tables S5**, **S6**) in a biplot with 95% confidence ellipses of concentration for the four series.

Morphometric and phylogenomic data were integrated to investigate the patterns of morphological evolution in *Epimedium* sect. *Diphyllon*. We used the phylomorphospace approach (Sidlauskas, 2008) implemented in the R package phytools (Revell, 2012) to reconstruct the evolutionary history of morphological diversification. A backbone input tree from the IQ-TREE analysis based on the *de novo* dataset was selected, and one individual from each taxon was used. The tree included 35 species from *Epimedium* sect. *Diphyllon* and one species from *Epimedium* sect. *Macroceras*. As previous AFLP (Zhang et al., 2014b) and cpDNA (Liu, 2021) results suggest that the sister lineage of *Epimedium* sect. *Diphyllon is E. elatum* from sect. *Polyphyllon*, we used the morphological traits of *E. elatum* rather than those of *E. koreanum* in the ancestral state analysis to estimate the ancestral character state of the section. The phylogenetic tree was then projected onto the multivariate morphospace defined by the first two principal components from the reproductive (**Supplementary Tables S7**, **S8**) and vegetative traits (**Supplementary Tables S9**, **S10**) to investigate the patterns of morphological evolution.

### 2.5 Evolution of morphological traits

To investigate particular morphological characters and phenotypic convergence, we conducted ancestral state reconstructions (ASRs) in Mesquite 3.70 (Maddison and Maddison, 2021) using the same phylogenetic tree used for the morphospace analysis. As previous AFLP (Zhang et al., 2014b) and cpDNA (Liu, 2021) results suggest that the sister lineage of *Epimedium* sect. *Diphyllon is E. elatum* from sect. *Polyphyllon*, we used the morphological traits of *E. elatum* rather than those of *E. koreanum* in the ancestral state analysis to estimate the ancestral character state of the section. Four characters that represent reproductive variation across the section were analyzed: (1) flower size (small and large); (2) flower shape (Type 1: lamina flat, obovate, with a slight median nectariferous furrow without spur or a saccate spur; Type 2: lamina rounded and conspicuous but expanded outwards at the base into an elongated spur usually longer than the inner sepal; Type 3: lamina reduced to a rim around the mouth of the elongated basally swollen spur, which is longer than the inner sepal; Type 4: lamina reduced to a rim around the mouth of the slipper-like cylindrical spur, which is shorter than the inner sepal; Type 5: both lamina and spur are reduced, forming a minute pouch); (3) inner sepal color (white, yellow, red, and purple); (4) spur color (white, yellow, red, purple, and brown).

### 2.6 Divergence time estimation

We estimated a time-calibrated tree using penalized likelihood as implemented in treePL (Smith and O’meara, 2012). When working with phylogenies obtained from a massive amount of data, such as those produced by GBS, treePL software is appropriate for estimating divergence times (Zheng and Wiens, 2015). The IQ-TREE tree obtained from the *de novo* dataset was used as input. The tree was pruned to include a single, arbitrarily chosen individual of each species. Calibration ages were based on the results of the dating analysis of the plastid genome (Guo et al., 2021). The root node between the outgroup and *Epimedium* sect. *Diphyllon* with a maximum age of 2.11 million years ago (Ma).

### 2.7 Introgression and reticulate evolution

Inferences of reticulate events were run in the Julia package PhyloNetworks (Solís-Lemus et al., 2017) using the SNaQ method (Solís-Lemus and Ané, 2016), which performs maximum pseudolikelihood estimation of phylogenetic networks from a list of unrooted gene trees or a table of quartet concordance factors (CFs) of 4-taxon trees (quartets) under multispecies coalescence, incorporating ILS and reticulation events. The CFs were used to estimate a semi-direct species network with estimated reticulation events and γ-values (inheritance probabilities of ancestral contributions to hybridization events). This strategy accelerates the likelihood computation by considering subsets of quartets instead of simultaneously calculating the full likelihood across all taxa. SNP2CF (Olave and Meyer, 2020) was used in R to convert the unlinked SNP PHYLIP file of ipyrad into a CF table. Afterward, the phylogenetic networks were reconstructed using PhyloNetworks. The SNaQ function used the CF table and ran 50 independent analyses with the number of reticulation events (h) ranging from zero to nine, using the gene tree as the starting topology for each h value. The best-fit number of reticulation events was inferred by plotting the likelihood scores for each run and observing the point at which additional reticulations did not improve the pseudolikelihood score (Solís-Lemus et al., 2017). We then used the pseudoreplication approach in SNP2CF to create a CF table with credibility intervals that were used in the PhyloNetworks Bootsnaq command to conduct 100 bootstrap replications to estimate the support for the inferred inheritance probabilities.

## 3 Results

### 3.1 Phylogenetic analyses

For the 57 samples sequenced for GBS, we obtained a total of 895 million reads with 15.7 million reads per sample on average. The final data from *de novo* assembly contained 88135 parsimony-informative sites with a total length of 820547 bp (73.0% missing data) and 10331306 bp (73.5% missing data) for the Denovo_SNP and Denovo datasets, respectively; for the reference-based data there were 49034 parsimony-informative sites with a total length of 931802 bp (76.4% missing data) and 9039893 (77.4% missing data) for the Ref_SNP and Ref datasets, respectively.

**Figure 2:**
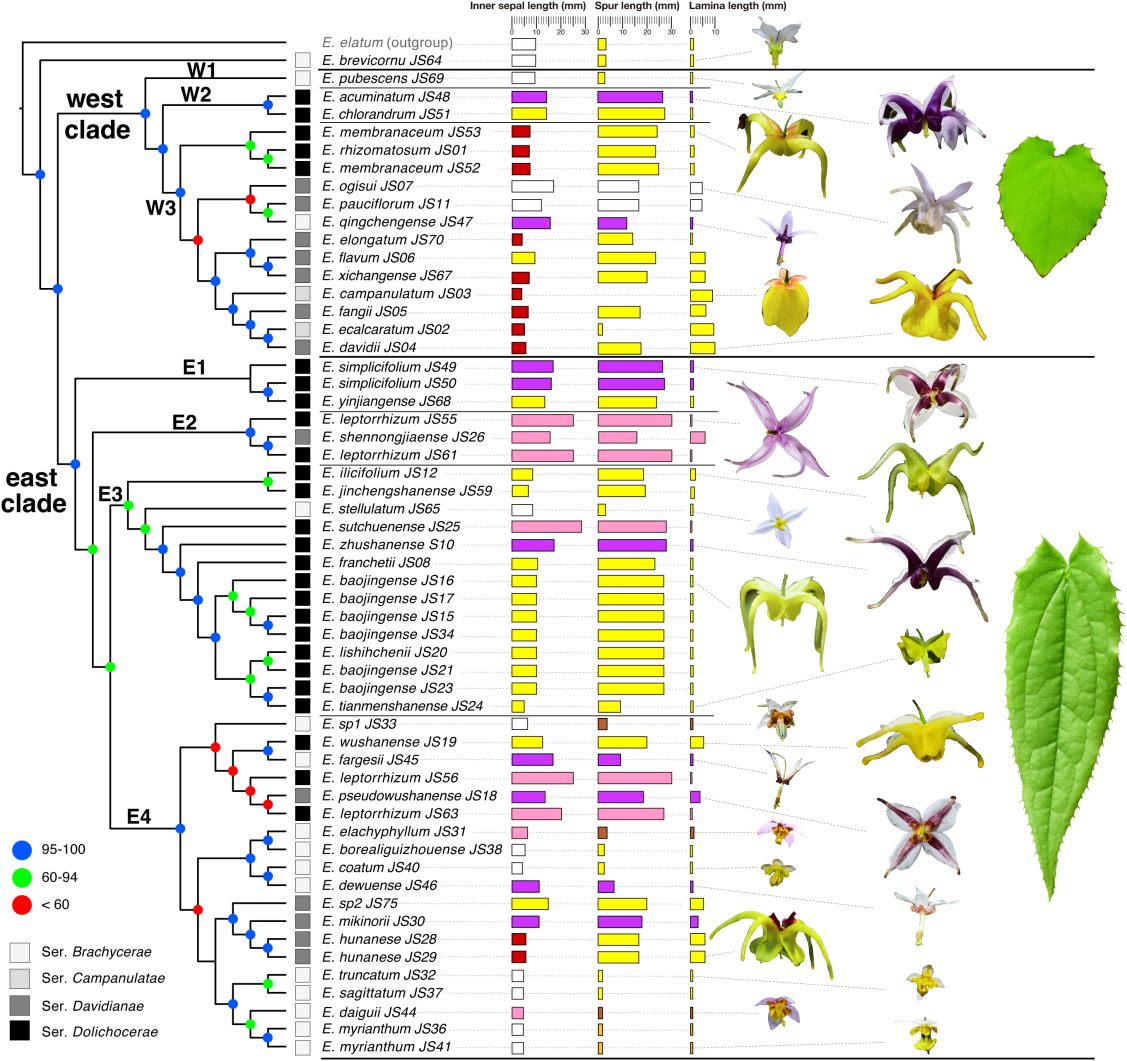
Phylogenetic relationships and morphological diversity of *Epimedium* sect. *Diphyllon* in the east Asia. Phylogenetic tree obtained from the IQ-TREE method based on the denovo GBS dataset, and node support is indicated with colored circles. The four series of sect. *Diphyllon* is indicated by different colored quadrate. Morphological traits are shown for the taxa, including mean values of inner sepal length, spur length, and lamina length. Flowers and leaves of sect. *Diphyllon* are shown.

Based on the four GBS datasets, phylogenetic analyses using the RAxML, IQ-TREE, and ExaBayes approaches produced 12 similar topologies with solid support (**Supplementary Figures S1A-C**, **S2A-C**, **S3A-C**, **S4A-C**). A phylogenetic tree based on the IQ-TREE method using the *de novo* GBS dataset with fully resolved and well-supported interspecific relationships was presented in **Figure 2**. All species of *Epimedium* sect. *Diphyllon* were grouped into two clades—a west clade and an east clade—except for *E. brevicornu*, which was discovered as a basal lineage of *Epimedium* sect. *Diphyllon* (**Supplementary Figure 2**, **Supplementary Figures S1A-C**, **S2B**, **S3A, C**), or a sister lineage to either the west or (**Supplementary Figures S2A, B**, **S3B**, **S4A-C**) east clades (**Supplementary Figure S1C**).

Seven subclades with strong support were identified using concatenated approaches. The west clade was divided into three subclades (W1, W2, and W3), with *E. pubescens* as the first diverging lineage (W1), and the remaining species were grouped into two subclades (W2 and W3) (**Figure 2**, **Supplementary Figures S1A-C**, **S2A-C**, **S3A-C**, **S4A-C**).

The east clade was divided into four subclades (E1, E2, E3, and E4) (**Figure 2**; **Supplementary Figures S1A-C**, **S2A-C**, **S3A-C**, **S4A-C**). E1 contains two species (*E. yinjiangense* and *E. simplicifolium*) and is supported as a distinct evolutionary lineage by both Denovo and Denovo_SNP datasets (**Figure 2**, **Supplementary Figures S1A-C**, **S2A-C**). However, the two reference-based datasets present different topologies, with *E. jinchengshanense* nested within them (**Supplementary Figures S3A-C**, **S4A-C**). E2 includes two species (*E. leptorrhizum* and *E. shennongjiaense*), and all topologies support it as a monophyletic lineage (**Figure 2**, **Supplementary Figures S1A-C**, **S2A-C**, **S3A-C**, **S4A-C**). E3 is recovered as a monophyletic lineage from the Denovo and Denovo_SNP datasets (**Figure 2**, **Supplementary Figures S1A-C**, **S2A-C**), but paraphyletic from the reference-based datasets (**Supplementary Figure S3A-C**, **S4A-C**). Finally, the remaining species in E4, such as *E. fargesii*, *E. hunanense*, and *E. myrianthum*, are supported as monophyletic lineages across all topologies (**Figure 2**; **Supplementary Figures S1A-C**, **S2A-C**, **S3A-C**, **S4A-C**).

SVD quartet analyses produced topologies similar to those of the concatenated gene trees (**Supplementary Figures S1D**, **S2D**, **S3D** & **S4D**). Two slightly different species-level topologies were generated from the Denovo and Denovo_SNP datasets (**Supplementary Figures S1D**, **S2D**). In the first topology, an individual of *E. leptorrhizum*_JS63 from Suiyang, Guizhou, was found to be closely related to *E. jinchengshanense*, as it was recovered as a sister lineage. In the second topology, a cluster of *E. wushanense* and *E. fargesii* was found to be a sister lineage to E3 (**Supplementary Figures S1D**, **S2D**). The SVD species tree generated from the Ref dataset supports *E. brevicornu* as the basal species in *Epimedium* sect. *Diphyllon*, with *E. jinchengshanense* as a sister species to E1 (**Supplementary Figure S3D**). The SVD species tree generated from the Ref_SNP dataset was similar to the Ref tree at the subgroup level; however, *E. brevicornu* is suggested as the basal species in the east clade (**Supplementary Figure S4D**).

### 3.2 Patterns of morphological evolution

**Figure 3:**
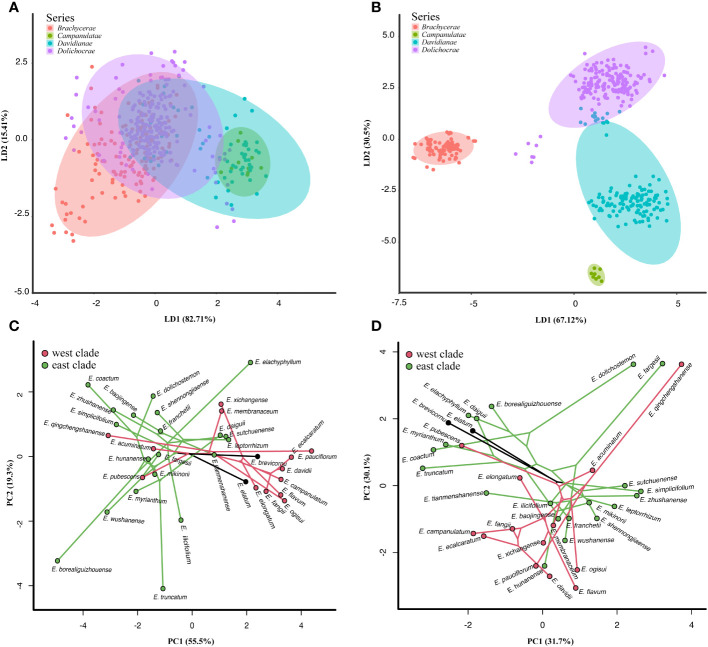
Morphometric analyses of *Epimedium* sect. *Diphyllon*. **(A)** Morphometric analyses on DFA of the vegetative traits (737 individuals, 42 taxa and one unknown species). **(B)** Morphometric analyses based on DFA of the reproductive traits (699 individuals, 41 taxa one unknown species). **(C)** Phylomorphospace of vegetative traits defined by a backbone tree with 35 species from IQ-TREE analysis based on the denovo GBS dataset. Each species is represented by the mean values. **(D)** Phylomorphospace of reproductive traits defined by a backbone tree with 35 species from IQ-TREE analysis based on the denovo GBS dataset. Each species is represented by the mean values of the coordinates.

Morphometric analyses were performed based on 11 vegetative traits from 737 individuals of 42 taxa and one unknown species from *Epimedium* sect. *Diphyllon*. The results showed that the individuals from the four series identified by Stearn (2002) were mixed together (**Figure 3A**). Biplots of sites and variables depicted differences between clusters, in particular vegetative morphometric characters for leaf texture, the ratio of middle leaflet length to width, and sawtooth density. The first two LD coordinates (explaining 98.12% of the variance) depicted a complex morphospace (**Figure 3A**). The results are based on 11 reproductive characters from 699 individuals of 42 taxa and one unknown species from *Epimedium* sect. *Diphyllon* revealed that the morphometric differentiation is consistent with the taxonomic treatment by Stearn (2002) (**Figure 3B**). Biplots of sites and variables depicted differences between clusters, in particular, flower morphometric characters for lamina presence or absence, inner sepal color, spur color, and spur reflection. The first two LD coordinates (explaining 97.62% of the variance) depicted a complex morphospace (**Figure 3B**).

Combined with phylogenetic and morphometric data, the phylomorphospace revealed distinct morphological patterns in the east and west clades within *Epimedium* sect. *Diphyllon.* The phylomorphospace, based on vegetative data, indicates the ancestral leaf shape of *Epimedium* sect. *Diphyllon* appears to have broadly ovate leaflets (**Figure 3C**). *Epimedium brevicornu*, the basal species of *Epimedium* sect. *Diphyllon*, has maintained vegetative traits that closely resemble those of the common ancestor of the *Epimedium* sect. *Diphyllon.* In contrast, the other species underwent significant morphological changes in multiple directions. In the phylomorphospace plot, the species of the west clade broadly occurred on the right side of the diagonal and displayed broad ovate to ovate leaflets (e.g., *E. davidii*, *E. elongatum*, and *E. xichangense*). In contrast, the species of the east clade are mostly distributed on the left side of the diagonal and displayed ovate-to-lanceolate leaflets. Additionally, convergence in vegetative traits is suggested by the close coordinates in different phylogenetic clades, as seen in *E. pubescens*/*E. myrianthum* with ovate leaflets.

The phylomorphospace defined by the reproductive traits (**Figure 3D**) suggests the floral ancestor state of *Epimedium* sect. *Diphyllon* appears to be a small flower with an inconspicuous spur and distinct lamina. This basic floral shape was retained by *E. brevicornu, E. pubescens*, and other small flowering species (Type 5). However, other species have undergone vast morphological shifts in different directions, even in sister-species pairs, including *E. acuminatum/E. chlorandrum* (purple corolla vs. yellow corolla), *E. membranaceum*/*E. pauciflorum* (yellow corolla, petals without lamina vs. white corolla, petals with lamina)*, E. elongatum/E. xichangense* (petals without lamina vs. petals with lamina) and *E. fangii/E. ecalcaratum* (petals with spur vs. flat petals), *E.leptorrhizum/E.shennongjiaense* (petals without lamina vs. petals with lamina)*, E. wushanense/E. fargesii* (large flowers, short stamens vs. small flowers, prolonged stamens)*, E. hunanense/E. mikinorii* (corolla yellow and significant lamina vs. corolla purple and medium-sized lamina), and *E. coatum/E. dolichostemon* (short stamen vs. prolonged stamen). The mapping of phylogenetic relationships onto geographic distributions revealed pairs of morphologically divergent sister species found in close proximity to one another. In contrast, convergence is suggested by the neighborhood in the morphospace of non-closely related species pairs (**Figure 3D**). Large flowers, yellow-dominated corollas, short sepals, large nectar spurs, and significant laminae have evolved independently in *E. hunanense* and *E. davidii* (Type 1). Overall, semblable floral morphologies, large flowers, broadly obovate inner sepals, and large nectar spurs without lamina have evolved independently in *E. simplicifolium* and *E. acuminatum* (Type 2). The floral traits of large flowers, yellow corollas, large nectar spurs, and those without lamina have evolved independently in *E. membranaceum* and *E. baojinense* (Type 2).

**Figure 4:**
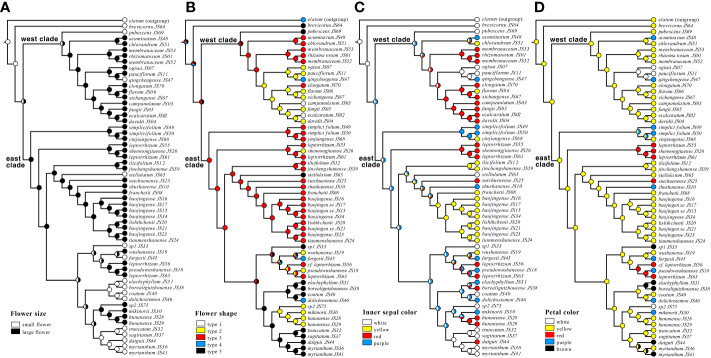
Patterns of morphological evolution of the *Epimedium* sect. *Diphyllon*. **(A)** Ancestral states were reconstructed from flower size, **(B)** flower shape, **(C)** inner sepal color, **(D)** petal color.

MP reconstructions facilitated the unambiguous inference of multiple shifts in flower size, flower type, inner sepal color, and spur color (**Figure 4**). The ancestral flowers were small (**Figure 4A**). The character evolution of the five flower types also suggests that the small flowers of Types 4 and 5 were in the ancestral state (**Figure 4B**). Large flowers and long spurs have independently evolved multiple times in both the west and east clades. This phenomenon was also observed in flower color (**Figures 4C, D**). The plesiomorphic state should be white, with diversification into yellow, red, pink to purple flower colors (**Figure 4C**). For petals or spurs, yellow color was the ancestral state; therefore, flower colors have evolved multiple times from yellow to purple and white in the west clade and from yellow to purple, red, and brown in the east clade (**Figure 4D**).

### 3.3 Divergence time estimation

**Figure 5:**
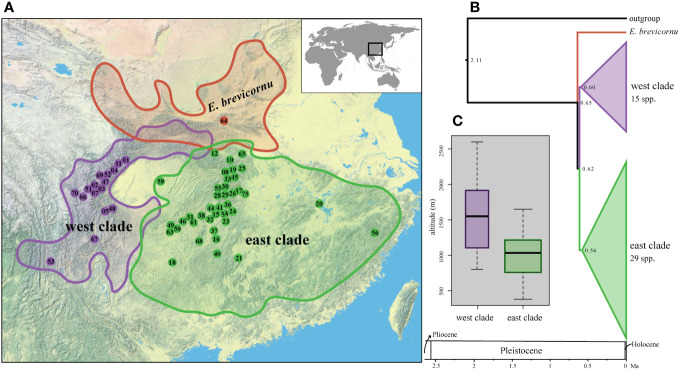
Phylogenetic and spatiotemporal context of the *Epimedium* sect. *Diphyllon* radiation in EA. **(A)** Geographic distribution of three lineages within *Epimedium* sect. *Diphyllon*. **(B)** Divergence times estimated using treePL approach from IQ-TREE analysis based on denovo dataset. **(C)** Boxplot of altitude difference between west clade and east clade.

Time estimation suggests that the extant species of *Epimedium* sect. *Diphyllon* diverged in the late Pleistocene at about 0.84 (0.57–1.25) Ma with *E. brevicornu* as the first split lineage (**Figure 5**). The divergence age between the east and west clades was estimated to be 0.77 (0.43–1.16) Ma (**Figure 5**).

### 3.4 Phylogenetic network analysis

**Figure 6:**
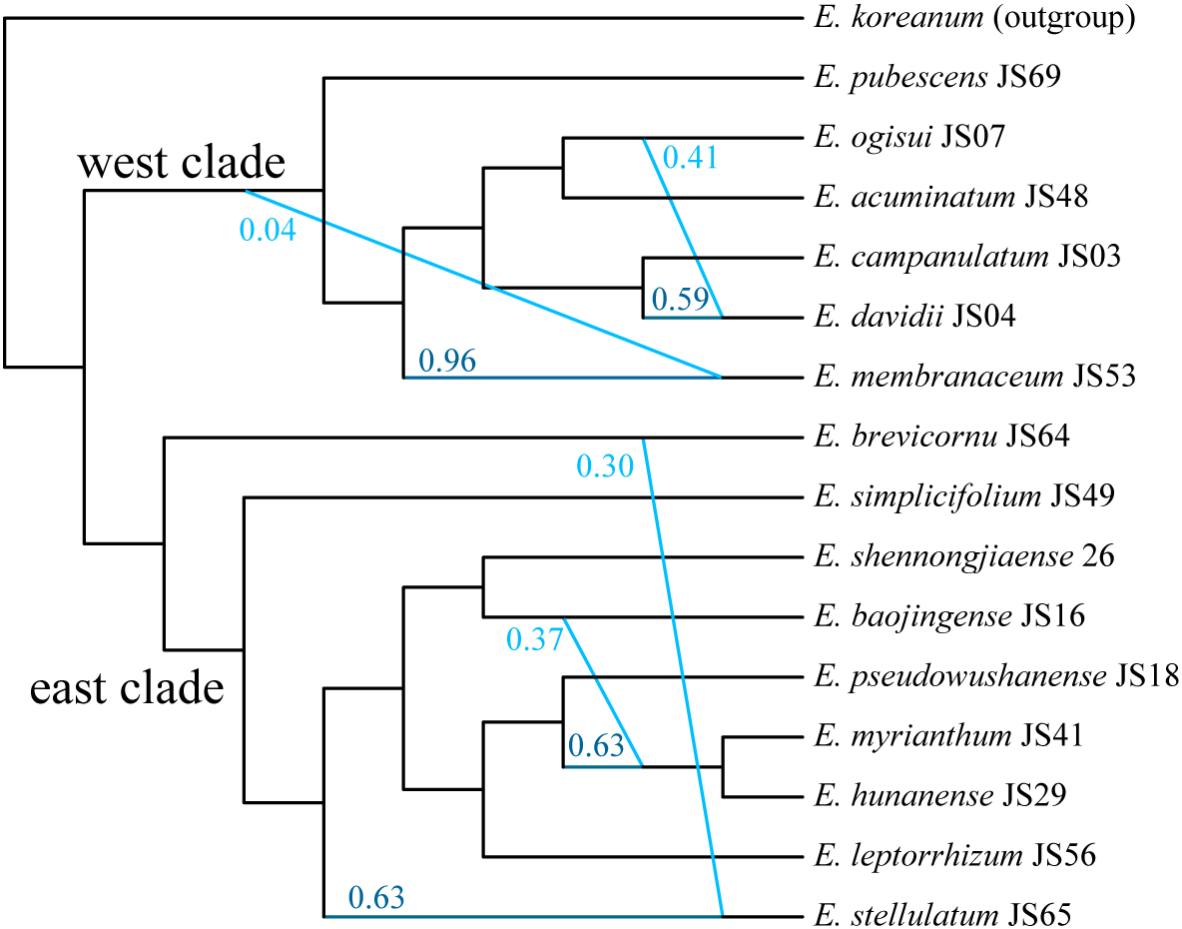
Phylogenetic network of *Epimedium* sect. *Diphyllon* estimated from SNaQ analysis with hmax = 5.

We conducted SNaQ analysis using 16 representative species to reconstruct a phylogenetic network within *Epimedium* sect. *Diphyllon* in EA. Based on the log pseudo-likelihood scores, the optimal network model was identified as h=5 (**Supplementary Figure S5**). The phylogenetic network generated by SNaQ showed topologies similar to those of the concatenated and coalescent analyses, supporting the differentiation between the west and east clades. This network illustrates the rapid radiation of *Epimedium* sect. *Diphyllon* species with four hybridizing edges: a) *E. davidii* is a hybrid species between *E. ogisui* (γ = 0.414) and *E. campanulatum* (γ = 0.586); b) *E. membranaceum* shows minor genetic introgression from the common ancestor of the west clade (γ = 0.0363); c) *E. stellulatum* has ca. 30% genomic exchange from *E. brevicornu* (γ = 0.294); and d) the *E. myrianthum*–*E. hunanense* lineage has an introgressed origin between *E. baojinense* (γ =0.372) and *E. pseudowushanense* (γ =0.628) (**Figure 6**).

## 4 Discussion

### 4.1 Robust phylogenetic relationships within *Epimedium* sect. *Diphyllon*

Phylogenetic relationships among *Epimedium* sect. *Diphyllon* remains unclear despite the use of numerous conventional molecular markers, such as AFLP, or plastid genome data (Sun et al., 2005; Zhang et al., 2007; De Smet et al., 2012; Zhang et al., 2014b; Guo et al., 2021). Phylogenomic approaches can help reconstruct speciation patterns and processes in detail, particularly in cases where conventional phylogenetic markers fail, as demonstrated for Galapagos giant daisies (Fernández-Mazuecos et al., 2020). Techniques based on high-throughput genomic sequencing, such as GBS, can generate genome-wide phylogenetic markers, providing unparalleled opportunities to test competing evolutionary hypotheses (Elshire et al., 2011; Qi et al., 2015; Fernández-Mazuecos et al., 2018). After generating a GBS library and testing both *de novo* and reference-based sequence dataset assemblies with multiple analytical analyses (**Supplementary Figures S1**-**S4**), the optimal combination produced a phylogenetic tree with fully resolved and well-supported interspecific relationships (**Figure 2**). Trees based on coalescent methods generally had lower support values, although the evolutionary patterns derived from both approaches were largely consistent (**Supplementary Figures S1D**, **S2D**, **S3D** & **S4D**).

Our phylogenomic analyses based on the GBS data revealed a much clearer evolutionary relationship for *Epimedium* sect. *Diphyllon* than before, with average node supports of 93.86%/1/90.34% from ML/BI/SVD analyses, respectively (**Figure 2**; **Supplementary Figures S1**-**S4**, **Supplementary Table S11**). Except for the uncertain placement of *E. brevicornu*, all accessions were grouped into two well-supported clades (**Figure 2**; **Supplementary Figures S1**-**S4**). All the results of our phylogenetic trees showed that *E. brevicornu* is an early diverging lineage, but its phylogenetic position was unstable. Specifically, *E. brevicornu* was placed as a sister species to the remaining species of *Epimedium* sect. *Diphyllon* or a sister species to either the west or east clades (**Figure 2**; **Supplementary Figures S1**-**S4**). In a recent study by Guo et al. (2021), however, *E. brevicornu* HBXY is deeply nested in the west clade based on chloroplast genomic data. This species is characterized by small flowers, which may indicate the ancestral status and distribution of the *Epimedium* sect. *Diphyllon*.

The results show a comparable divergence pattern to the plastid genome phylogeny of Guo et al. (2021), albeit with relatively lower resolution in the chloroplast results. In line with the chloroplast genome findings of Guo et al. (2021), our nuclear genomic results also identify two groups. However, our nuclear genomic analysis offers a more detailed resolution of species relationships within each subgroup. Notably, most of the analyses strongly support the identification of three subgroups within the west clade and four subgroups within the east clade (**Figure 2**; **Supplementary Figures S1**-**S4**).

Except for *E. brevicornu*, topological differences were also detected between previous chloroplast and current GBS datasets for the other taxa within the section. *Epimedium pubuscens* is deeply nested within the west clade in the chloroplast results (Guo et al., 2021); however, it is consistently recognized as the first lineage of the west clade in the current nuclear genomic results (**Figure 2**; **Supplementary Figures S1**-**S4**). *Epimedium stellulatum* SXNZ in Guo et al. (2021) is placed in the west clade; however, our collection from Hubei belonged to the east clade (**Figure 2**; **Supplementary Figures S1**-**S4**). *Epimedium pauciflorum* SCMS belongs to the east clade in the study of Guo et al. (2021), while our collection from Sichuan, Maoxian, belonged to the west clade (**Figure 2**; **Supplementary Figures S1**-**S4**). These phylogenetic conflicts between different genomic data might have resulted from the low resolution of the chloroplast sequences or from hybridization or introgression between them. Further tests with more data are needed.

### 4.2 Biogeographic vicariance in the early divergence

A morphology-based taxonomic treatment by Stearn (2002) was rejected by our team and chloroplast results (Guo et al., 2021) that the four series defined by flower characters are not monophyletic (**Figure 2**; **Supplementary Figures S1**-**S4**). *Epimedium* sect. *Diphyllon* diverged into two clades, and species belonging to different series were mixed together in each clade (**Figure 2**; **Supplementary Figures S1**-**S4**). The west clade includes all four series: *Campanulatae*, *Davidianae*, *Dolichocerae*, and *Brachycerae*, whereas the east clade consists of *Davidianae*, *Dolichocerae*, and *Brachycerae* series (**Figure 2**; **Supplementary Figures S1**-**S4**). The taxonomic treatment was largely based on reproductive rather than vegetative characteristics (Stearn, 2002), which was also supported by the morphological analyses (**Figure 3B**). The vegetative data did not corroborate the taxonomic treatments when the four series were mixed together (**Figure 3A**). In contrast, the reproductive data provided evidence in favor of the taxonomic treatments that distinguished the species into four series (**Figure 3B**).

Despite the incongruence between morphological and phylogenetic relationships, there is strong evidence of geographic patterns within *Epimedium* sect. *Diphyllon*. The two main clades within *Epimedium* sect. *Diphyllon* broadly corresponds to the biogeographic distribution of southern EA (**Figure 5**). The west clade is composed of species mostly from western China, whereas those of the east clade are largely from eastern China. The division of the two clades into distinct biogeographic regions roughly matched the EA floristic division between the Sino–Japanese Forest and the Sino–Himalayan Forest subkingdoms as well as the environmental division between southwestern and eastern China (Wu and Wu, 1998), suggesting a close link between phylogenetic relationships, floristic evolution, and geographical changes in EA (Zhang et al., 2007). Geological differences, together with the formation and development of the Asian monsoon, may have been the main factors driving the evolution of EA flora (Zhang et al., 2007), as indicated by the divergence in *Epimedium* sect. *Diphyllon*. Moreover, these phytogeographical units are in agreement with the infraspecific relationships in *Spiraea japonica*, with two clades corresponding to the eastern and western divisions (Zhang et al., 2006). Major genetic subdivisions between the Sino–Himalayan and Sino–Japanese Forest subkingdoms have also been found in other plant taxa, such as *Ainsliaea* (Mitsui et al., 2008), *Cardiocrinum* (Lu et al., 2017), and across the East China Sea between Southeast China and Japan, such as *Kalopanax septemlobus* (Sakaguchi et al., 2012) and *Euptelea* (Cao et al., 2016).

Interestingly, we observed a morphological differentiation trend in leaf traits between the two clades (**Figure 3A**). The phylomorphospace results revealed that species of the west clade usually have membranous and broadly ovate leaflets, whereas those of the east clade have leathery, ovate, narrowly ovate, and lanceolate leaflets (**Figure 3A**). The phylogenetic separation of the two clades is related to the patterns of morphological differentiation in the two clades of *Epimedium* sect. *Diphyllon*. It appears that climatic and geological differences played important roles in the divergence of *Epimedium* sect. *Diphyllon* and probably initiated the leaf divergence of the two groups within southern EA (Cao et al., 2016).

### 4.3 Recent parallel and atavism evolution in flowers

Flower morphology is a critical feature that can be used to distinguish *Epimedium* species (Stearn, 2002). Based on the size relationship between the petals and inner sepals, the genus is divided into two groups (Stearn, 1993a; Stearn, 1993b). The small group usually consists of flat, slightly saccate, saccate, and short spurs with petals shorter than the inner sepals and < 1 cm in diameter, whereas the large-flowered group has long spurs with petals longer than the inner sepals and > 1 cm in diameter (**Figure 1**). Southern EA is the distribution and differentiation center of *Epimedium* sect. *Diphyllon* with both small and large flowers (Ying, 2002). The plesiomorphic state should be a relatively small flower, inner sepal larger than the petal, and petal with a short spur (Ying, 2002). Our data support the general hypothesis that the ancestral flower size should be small (**Figure 4A**) although a previous ancestral character reconstruction analysis suggests that a large flower with a long spur (large-flowered group) is the plesiomorphic state (Guo et al., 2021).

Stearn (2002) identified five types of flower shapes of *Epimedium* (**Figure 1**). Character evolution also suggests that the small flowers of types 4 and 5 are ancestral states (**Figure 4B**). The flowers independently evolved mainly in length and size, from small to large and back to small, in both the east and west clades (**Figure 4B**). It seems that parallel evolution is dominant in *Epimedium* sect. *Diphyllon*, as evidenced by the evolution of large flowers and long spurs in both west and east clades.

The evolution of spur length through pollinator shifts has been examined using phylogenetic evidence in recent studies (Whittall and Hodges, 2007; Boberg et al., 2014). Guo et al. (2021) suggested that the diversification of petal shape likely affected reproductive success and drove the evolution of *Epimedium*, leading to the evolution of the long spur to respond to pollinator shifts. Effective pollinators, such as bumblebees (*Bombus* spp.) and nectar-foraging bees, have been observed to be associated with species that have long spurs (Suzuki, 1984; Li et al., 2009). Bumblebees are a group of social bee species that form structured colonies, and the genus is composed of numerous species found widely in the Northern Hemisphere. The parallel evolution of large flowers with long spurs in both the west and east clades may be explained by the presence of similar bumblebees around flowers with long spurs in both regions.

A similar situation was also determined in the evolution of flower color (**Figures 4C, D**). The plesiomorphic state for flower color should be white, with diversification into yellow, red, and purple in inner sepals (**Figure 4C**). For petals or spurs, yellow is the ancestral state with a parallel shift from yellow to purple and white in the west clade or purple, red, and brown in the east clade (**Figure 4D**). Parallel evolution is common in plants and animals, and the repeated and similar large-scale morphological evolutionary trends of distinct lineages suggest that adaptation through natural selection (functional constraints) is the major cause of parallel evolution, which is a common phenomenon in extinct and extant lineages (Monnet et al., 2011). For example, enlarged flower heads have evolved repeatedly in *Artemisia*, which is considered helpful in attracting pollinators better (Tkach et al., 2008).

In biology, atavism is the modification of a biological structure, whereby an ancestral genetic trait reappears after having been lost through evolutionary changes in previous generations (Hall, 1984). Atavism probably occurred multiple times in both clades of southern EA *Epimedium*. Type 1, with flat petals, was assumed to be the ancestral type, which evolved into four other types: slightly saccate, saccate, short spur, and long spur (**Figure 1**). However, our results suggest that species with flat petals were deeply nested within the west clade (**Figure 4B**). The ancestral state for the petal shape of *Epimedium* should be flat without shifting to a saccate-like shape or spur (Stearn, 2002; Ying, 2002). The morphological traits of its sister genus *Vancouveria* and other genera of the family support this hypothesis although most *Epimedium* species have petals with short or long spurs (Zhang et al., 2007). Although there are very few species with flat petals, the phylogenetic results suggest that they are deeply nested within the tree, and character evolution analyses suggest that they have shifted back from species with spurs. The ancestral state of *Epimedium* sect. *Diphyllon* should be small flowers; however, a species with small flowers with short spurs have also evolved independently and multiple times in both clades (**Figure 4**).

The origin of *Epimedium* sect. *Diphyllon* dates back to c. 2.11 Ma in the early Quaternary (Zhang et al., 2007; Guo et al., 2021), and the divergence of the crown of *Epimedium* sect. *Diphyllon* is estimated to have taken place in the late Pleistocene epoch (**Figure 5**). It appears that the radiation of *Epimedium* sect. *Diphyllon* with parallel and atavism evolution of flowers is very recent, which is consistent with the relatively recent geological history of EA. The uplift of the Qinghai–Tibetan Plateau to its current altitude in southwest China, with allopatric differentiation of plants between southwest and east China, has been suggested to have a relatively recent history (Wen et al., 2014). Meta-analyses of seed plants from EA also suggest that the EA flora might be relatively young, with most of its clades originating from the Miocene (Chen et al., 2017).

### 4.4 Hybridization and introgression

*Epimedium* species occupy different areas in the wild and have evolved in geographical isolation. However, several species have overlapping ranges, which may result in hybridization (Stearn, 1995). We found strong evidence of extensive reticulation events during the diversification of *Epimedium* sect. *Diphyllon* in southern EA from the discordant phylogenetic relationships among gene trees, species trees (**Supplementary Figures S1**-**S4**), and especially, the SNaQ analysis (**Figure 6**). For example, the SNaQ results revealed that *E. davidii* is a hybrid species resulting from the interbreeding of *E. ogisui* (γ = 0.414) and *E. campanulatum* (γ = 0.586). The distribution of *E. davidii* overlapped with that of both *E. ogisui* and *E. campanulatum* in western China. *Epimedium davidii* exhibits intermediate characteristics; for example, its inner sepals (red, small, and lanceolate) and petal color (yellow) resemble those of *E. campanulatum*, while its petal form (with an elongated spur and obvious lamina) is similar to that of *E. ogisui*. *Epimedium stellulatum*, which has approximately 30% genomic exchange from *E. brevicornu* (γ = 0.294), has small flowers and broadly ovate leaflets that closely resemble those of *E. brevicornu*. It appears that *E. hunanense*, a species related to *E. baojinense* and *E. pseudowushanense*, exhibits intermediate traits between the two parental species. Specifically, its red-colored inner sepals and conspicuous lamina are similar to those of *E. pseudowushanense*, while its yellow-colored petals are similar to those of *E. baojinense*.

Evidence of extensive introgression has been found throughout the evolutionary history of *Epimedium* sect. *Diphyllon* (**Figure 6**) and reticulate evolution played significant roles in shaping the existing diversity in EA. Numerous cases have invaluably contributed to our understanding of the evolution of plant diversity in certain areas, with an increasing number of natural hybridization or introgression events have been identified in crops and wild species with genomic analyses recently (Rieseberg et al., 2007; Eaton and Ree, 2013; Penjor et al., 2016; Yang et al., 2016; Vargas et al., 2017; Morales-Briones et al., 2018; Lee-Yaw et al., 2019; Reichelt et al., 2021; Rose et al., 2021; Wang et al., 2021). For instance, significant interspecific introgression of *Pedicularis* sect. *Cyathophora* was identified using restriction site-associated DNA sequencing data (Eaton and Ree, 2013). Using the targeted enrichment approach to capture hundreds of nuclear loci and nearly complete plastome sequences, ancient and recent hybridization events in the genus *Lachemilla* were identified based on significant phylogenetic discordance and the results of species network analyses (Morales-Briones et al., 2018). Using phylogenomic data to explore the evolutionary history of the genus *Polemonium* showed that the cytoplasmic-nuclear discordance in this recently radiated genus was caused by both ILS and reticulate evolution (Rose et al., 2021). Other examples, such as Hawaii silversword, East Asian kiwifruit, and Iberian toadflax, have suggested that hybridization results in rapid speciation (Barrier et al., 1999; Liu et al., 2017; Fernández-Mazuecos et al., 2018).

The rampant introgressions in *Epimedium* sect. *Diphyllon* were probably caused by the lack of reproductive isolation. A series of experimental crosses provided strong evidence for an outbreeding system and a weak internal barrier to hybridization among the *Epimedium* species studied (Sheng et al., 2011). This is not surprising, considering that an outcrossing breeding system with strong self-incompatibility but high cross-compatibility between infraspecific and interspecific individuals is prevalent in *Epimedium* (Suzuki, 1983; Sheng et al., 2011). Interestingly, introgression events were mostly detected within the east or west clades, rarely between species from the two groups (**Figure 6**). Furthermore, hybridization events promote morphological diversity among species and obscure their evolutionary relationships, resulting in the extremely high diversity and complicated evolution of this lineage. The influence of hybridization on speciation and adaptive evolution should be further investigated.

## 5 Conclusions

**Figure 7:**
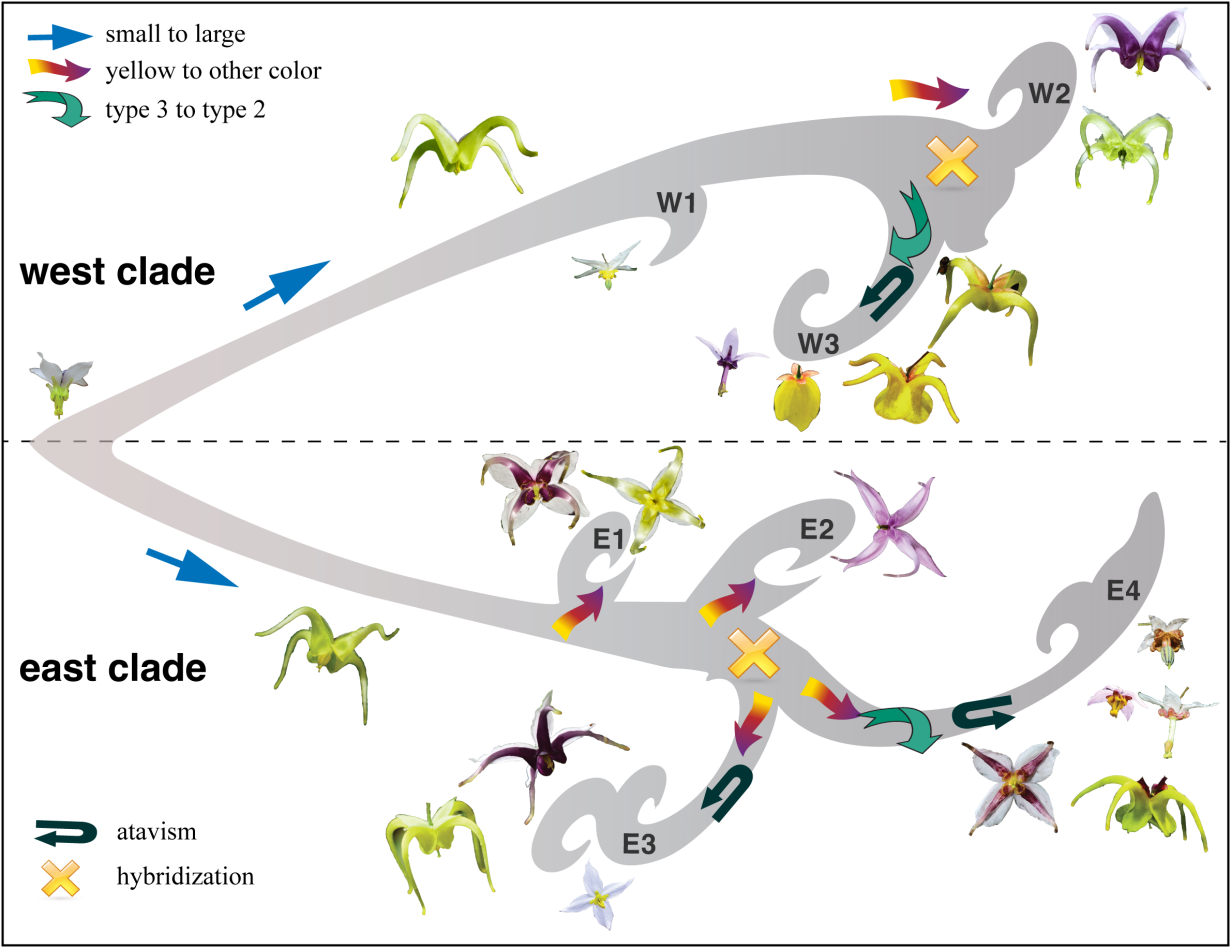
A schematic diagram showing the parallel-atavism-introgression pattern of *Epimedium* sect. *Diphyllon* radiated in southern EA.

Evolutionary radiations have long fascinated biologists since the publication of “On the Origin of Species” by Charles Darwin. EA is a natural floristic region with the highest biodiversity of flowering plants in the northern temperate zone. We conducted robust phylogenomic analyses to untangle the evolutionary patterns and processes of the barrenwort plant (*Epimedium*) during the course of its radiation mainly in southern EA. A parallel-atavism-introgression hypothesis (**Figure 7**) was referred to in explaining the radiation of plant diversity in southern EA, which provides a potential answer to the fundamental biological question of plant radiation on Earth. Moreover, massive genomic sequencing provided an essential framework for investigating evolutionary and ecological processes in the complex natural laboratories of EA, thereby advancing our understanding of plant radiation in biodiversity hotspots.

## Data availability statement

The datasets presented in this study can be found in online repositories. The names of the repository/repositories and accession number(s) can be found in the article/**Supplementary Material**.

## Author contributions

Z-LN, CZ, B-LG, and Q-RL designed the research. CZ collected the samples. CZ and B-LG identified all plant samples. Z-LN, CZ, RM, and YM performed experiments and lab work. Z-LN, CZ, and RM analyzed the data. All authors contributed to the article and approved the submitted version.
