# Supplementary figures and images for: Parallel evolution, atavism, and extensive introgression explain the radiation of Epimedium sect. Diphyllon (Berberidaceae) in southern East Asia
Source: Front Plant Sci. 2023 Oct 17;14:1234148. doi: 10.3389/fpls.2023.1234148 (PMC10616310; doi:10.3389/fpls.2023.1234148)

west  
clade

A

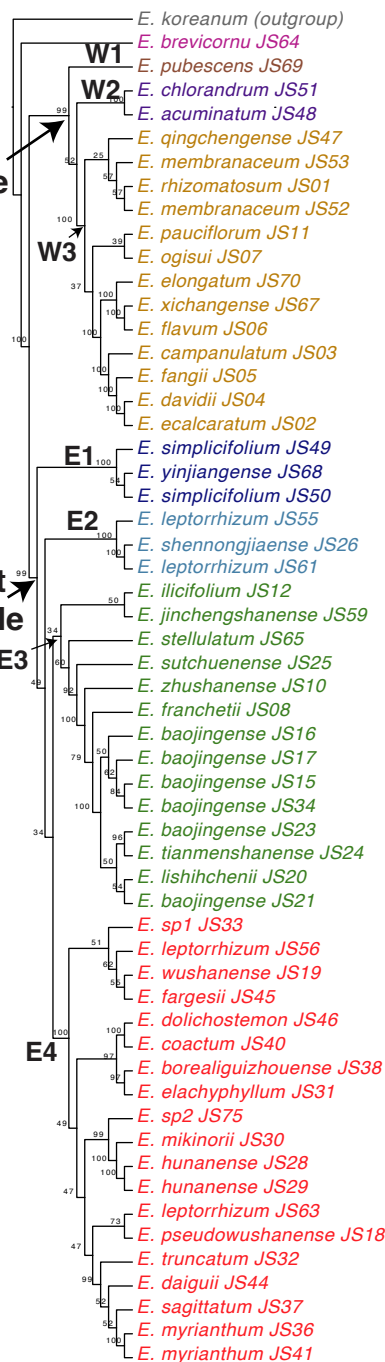

B

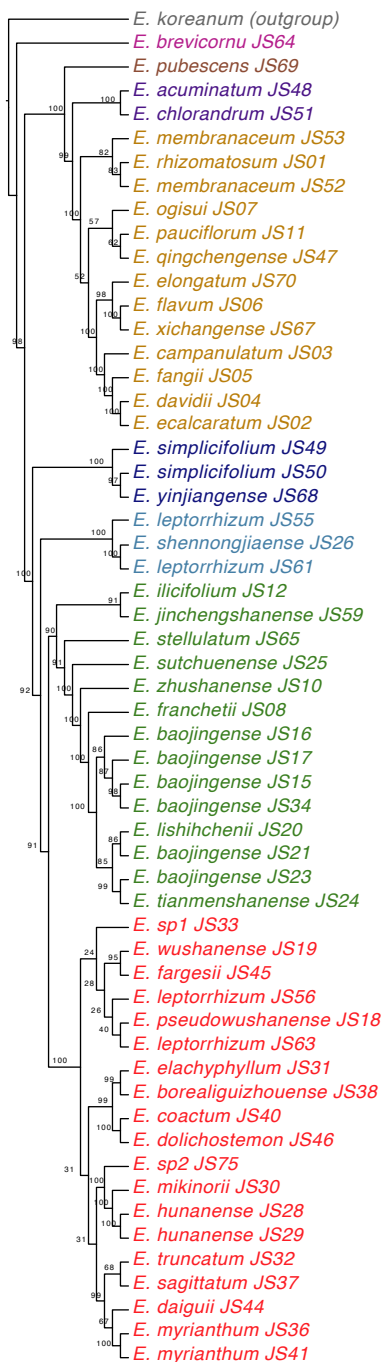

C

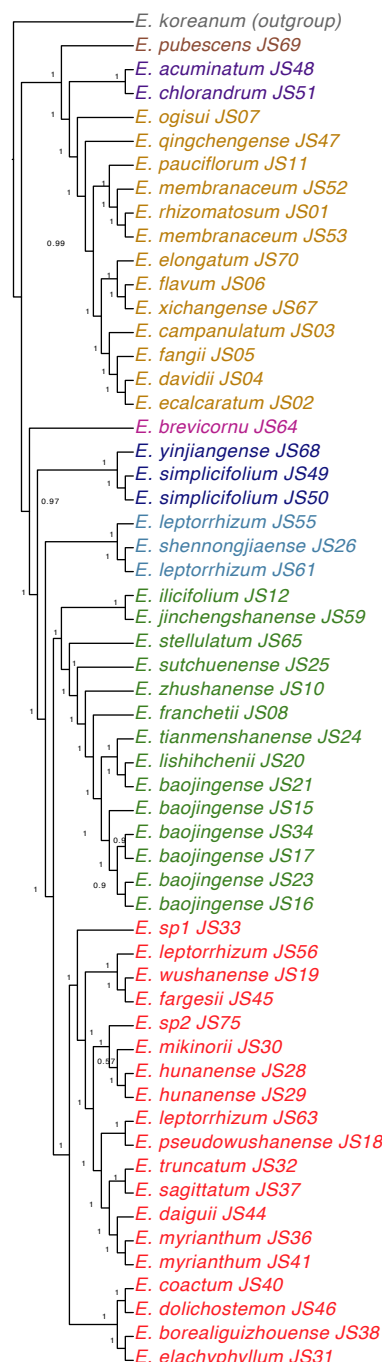

D

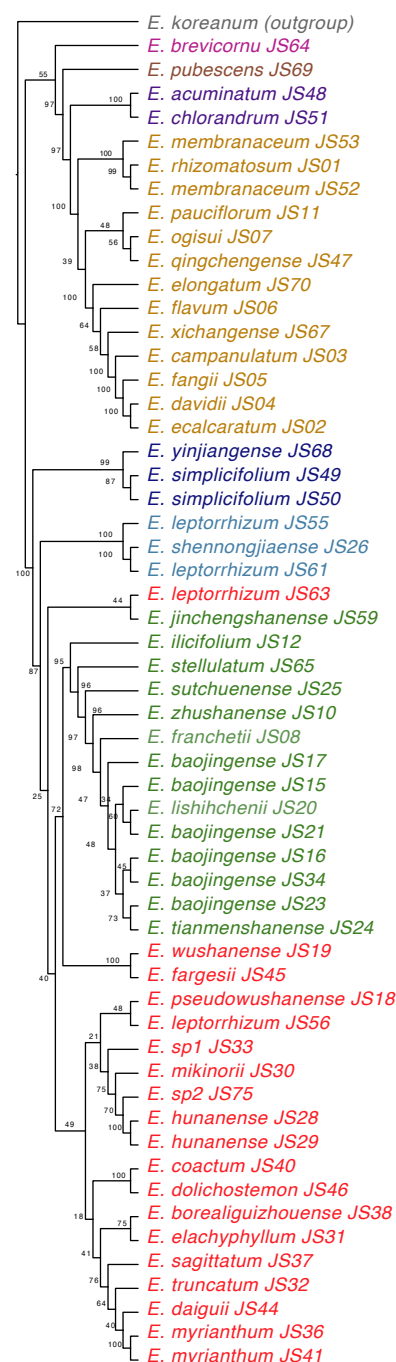

Supplement: Supplementary file 1 [file Image_1.pdf]

**A****B****C****D**west  
cladeeast  
clade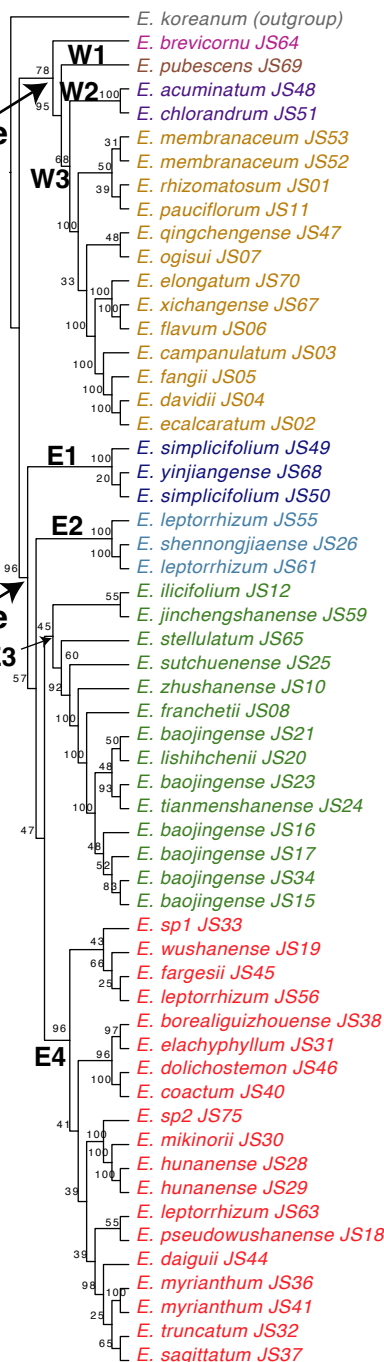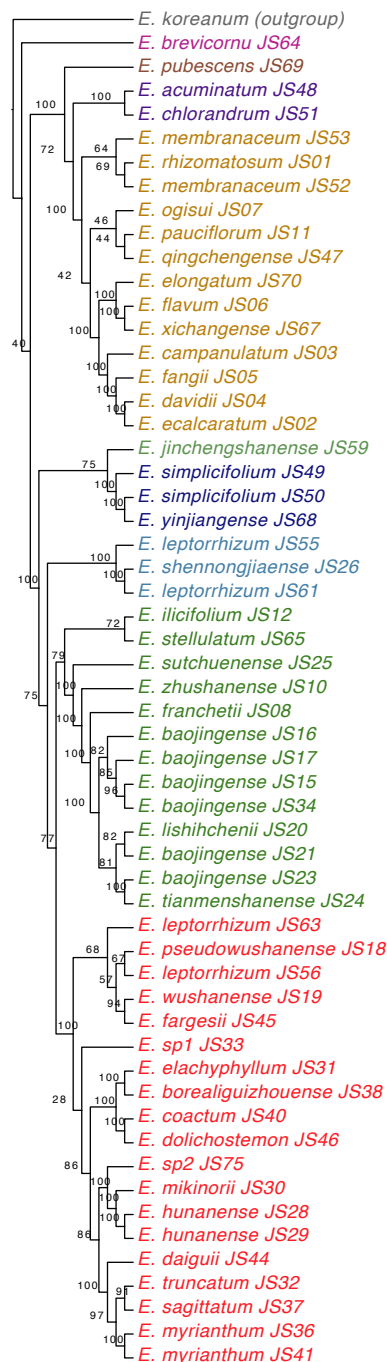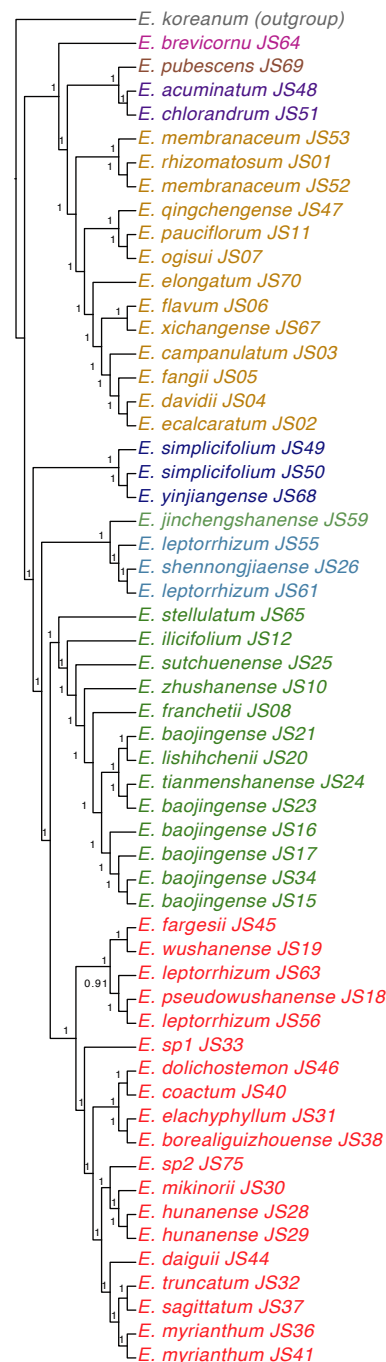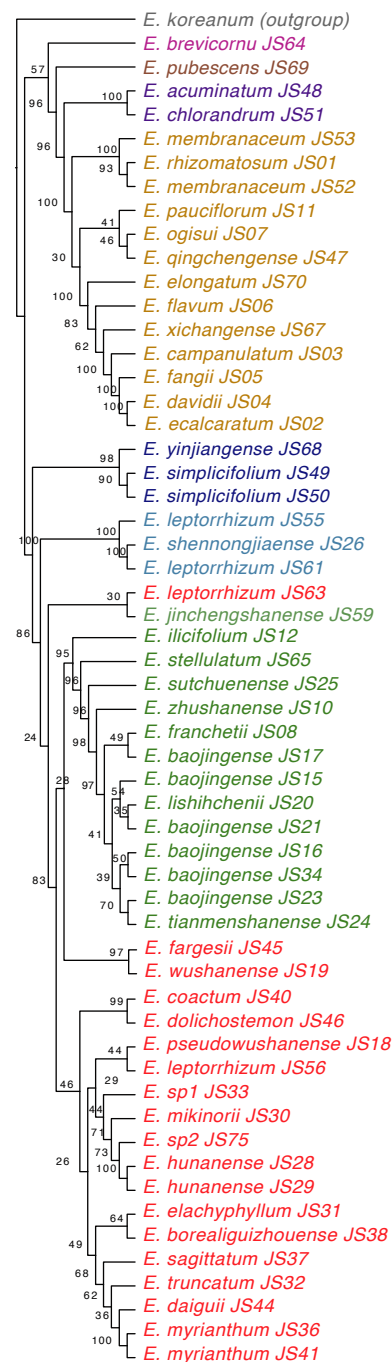

Supplement: Supplementary file 2 [file Image_2.pdf]

**A**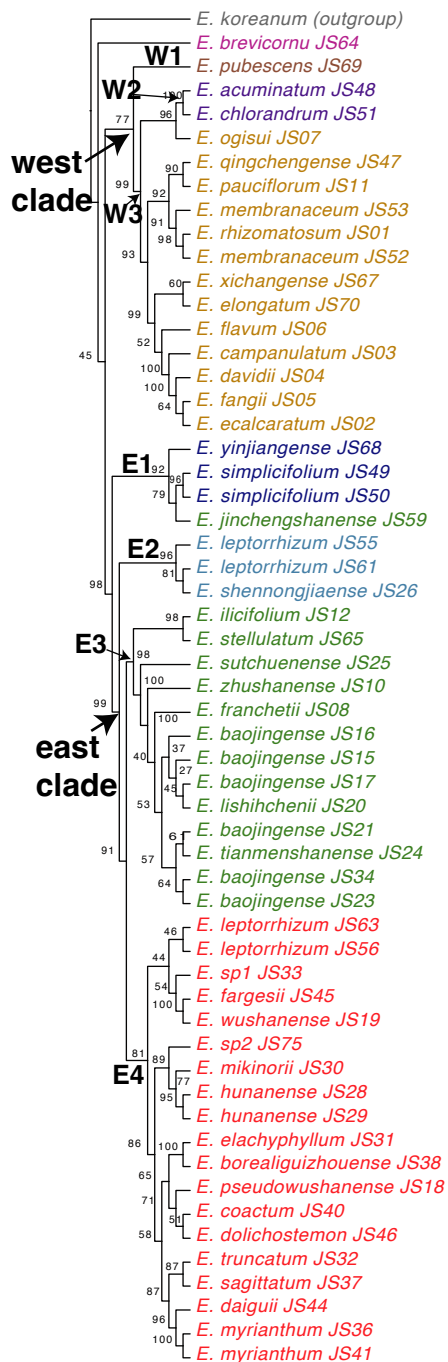**B**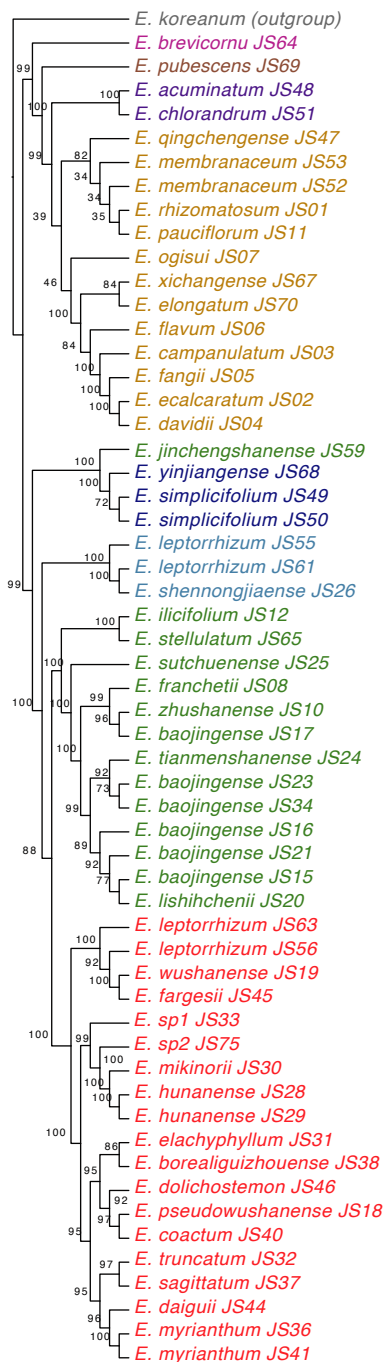**C**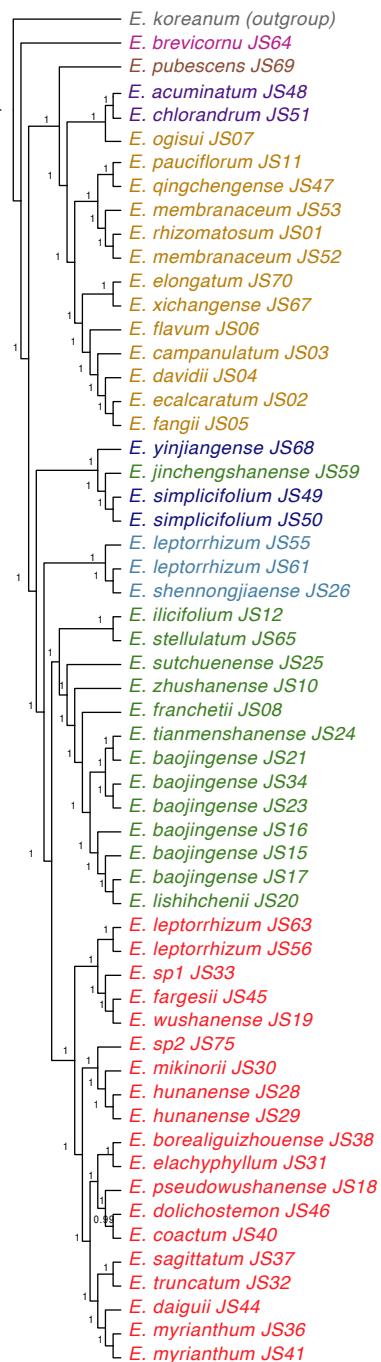**D**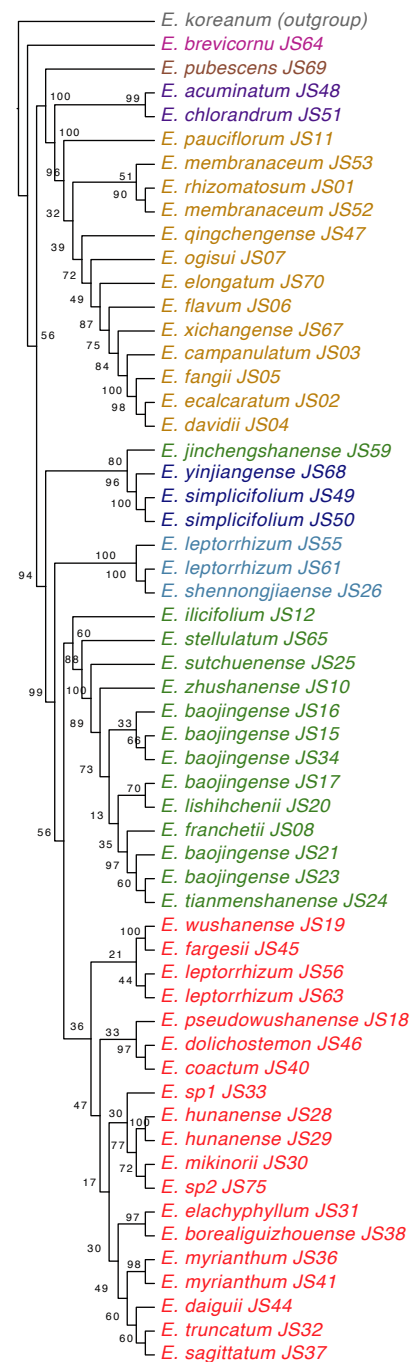

Supplement: Supplementary file 3 [file Image_3.pdf]

**A****B****C****D**west  
cladeeast  
clade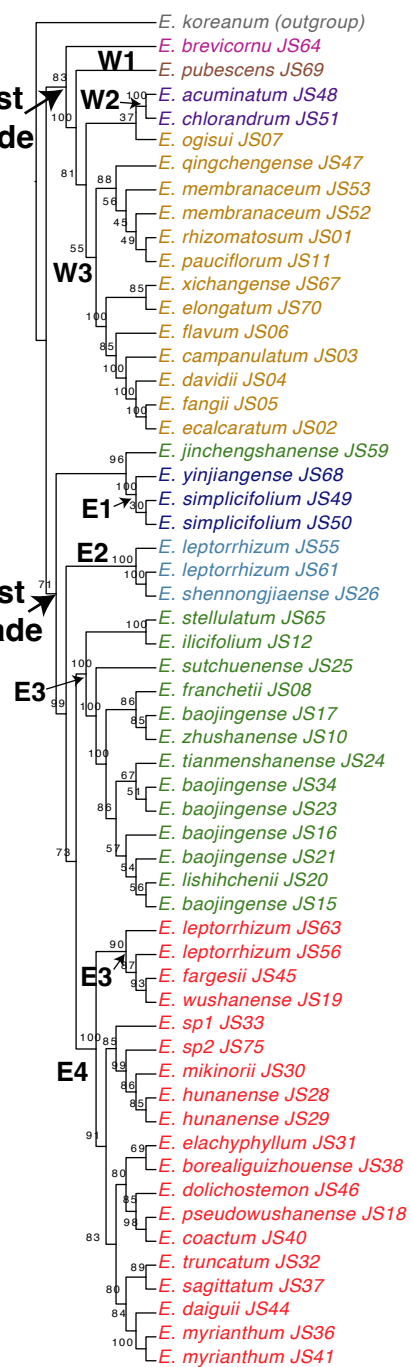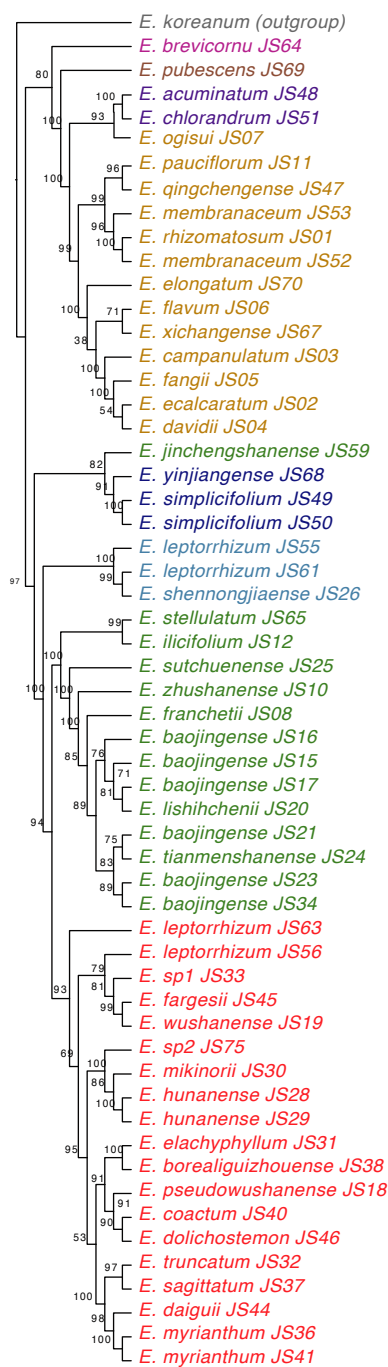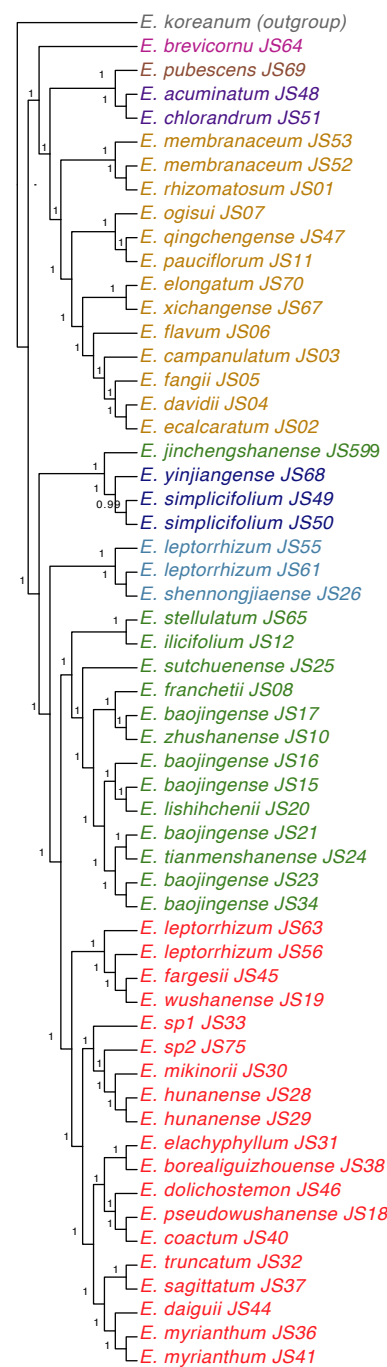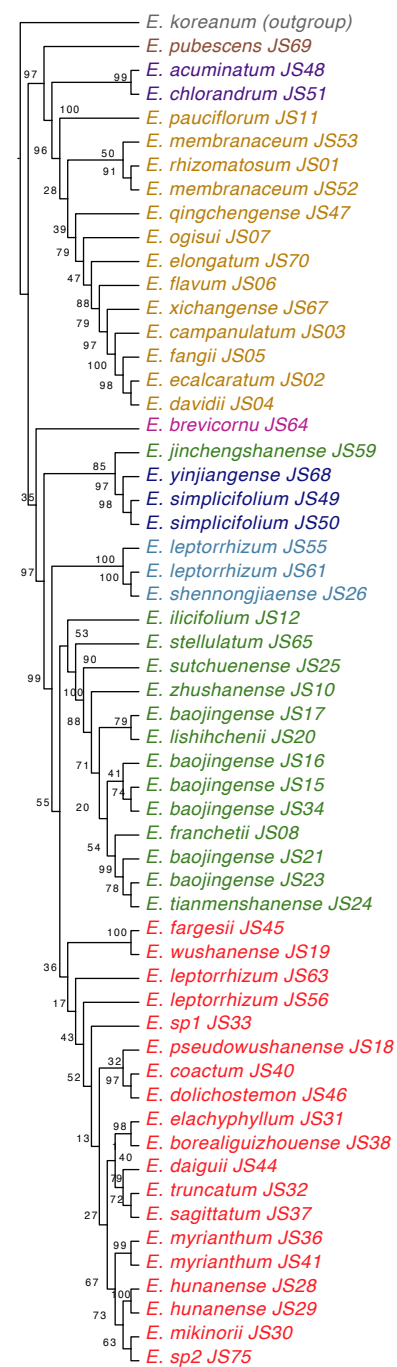

Supplement: Supplementary file 4 [file Image_4.pdf]

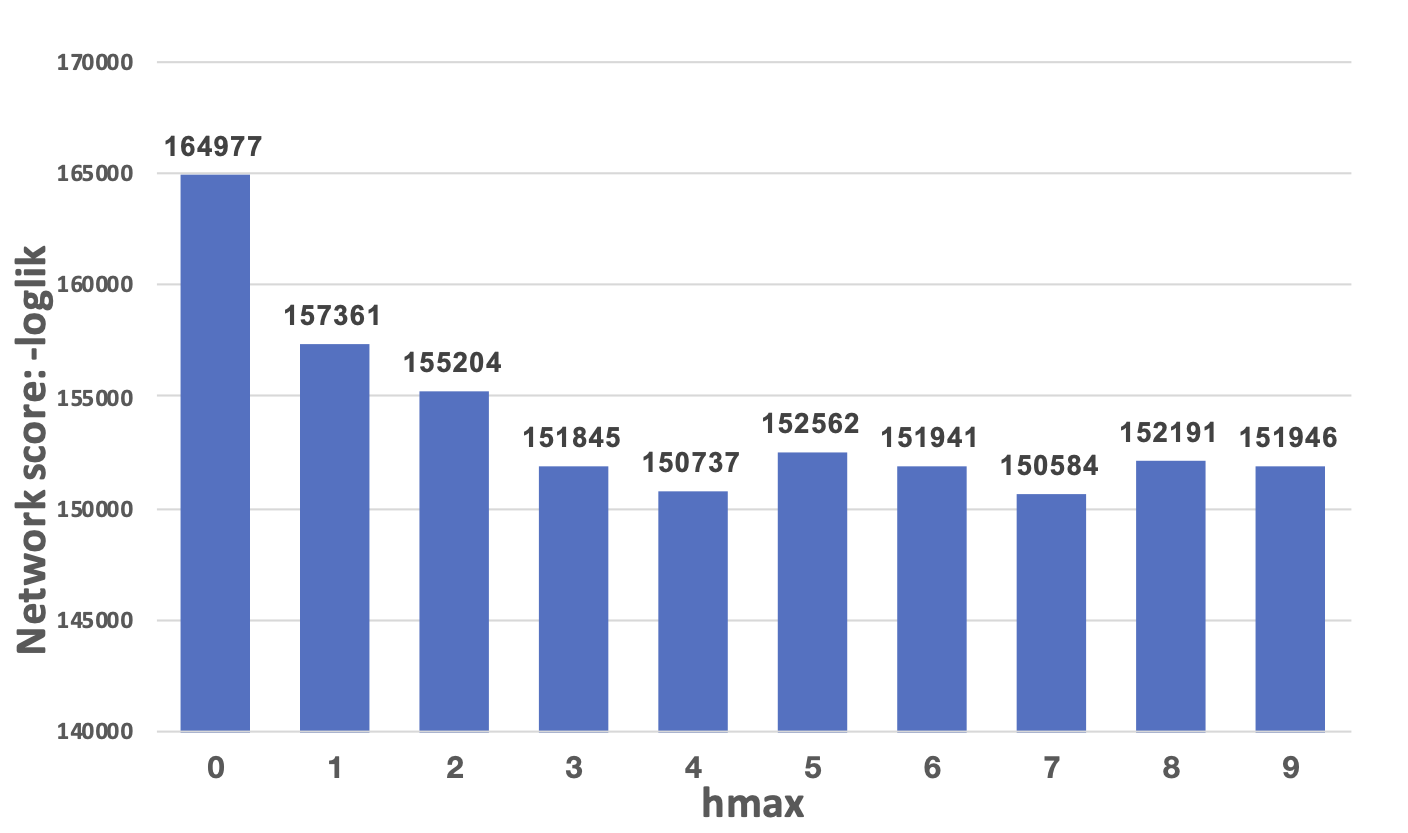

Supplement: Supplementary file 5 [file Image_5.tiff]
