# Supplementary material for: Parallel evolution, atavism, and extensive introgression explain the radiation of Epimedium sect. Diphyllon (Berberidaceae) in southern East Asia
Source: Front Plant Sci. 2023 Oct 17;14:1234148. doi: 10.3389/fpls.2023.1234148 (PMC10616310; doi:10.3389/fpls.2023.1234148)
Supplement: Supplementary file 7 [file Table_2.docx]

Supplementary Material

Parallel evolution, atavism, and extensive introgression explain the radiation of *Epimedium* sect. *Diphyllon* (Berberidaceae) in southern East Asia

Cheng Zhang, Ran Meng, Ying Meng, Bao-Lin Guo, Quan-Ru Liu*, Ze-Long Nie*

*** Correspondence:** Quan-Ru Liu: liuquanru@bnu.edu.cn; Ze-Long Nie: [niez@jsu.edu.cn](mailto:niez@jsu.edu.cn)

# Supplementary Figures and Tables

## Supplementary Figures

**Supplementary Figure S1.** Four phylogenetic analyses based on the GBS data from denovo assembly. **(A)** Gene tree obtained in RAxML from Denovo dataset. **(B)** Gene tree obtained in IQ-TREE from Denovo dataset. **(C)** Gene tree obtained in ExaBayes from Denovo dataset. **(D)** Species tree obtained in SVDquartets from Denovo dataset.

**Supplementary Figure S2.** Four phylogenetic analyses based on the GBS_SNPS data from Denovo assembly. **(A)** Gene tree obtained in RAxML from Denovo_SNPs dataset. **(B)** Gene tree obtained in IQ-TREE from Denovo_SNPs dataset. **(C)** Gene tree obtained in ExaBayes from Denovo_SNPs dataset. **(D)** Species tree obtained in SVDquartets from Denovo_SNPs dataset.

**Supplementary Figure S3.** Four phylogenetic analyses based on the GBS data from reference assembly. **(A)** Gene tree obtained in RAxML from Ref-based dataset. **(B)** Gene tree obtained in IQ-TREE from Ref-based dataset. **(C)** Gene tree obtained in ExaBayes from Ref-based dataset. (D) Species tree obtained in SVDquartets from Ref-based dataset.

**Supplementary Figure S4.** Four phylogenetic analyses based on the GBS_SNPS data from reference assembly. **(A)** Gene tree obtained in RAxML from Ref_SNP-based dataset. **(B)** Gene tree obtained in IQ-TREE from Ref_SNP-based dataset. **(C)** Gene tree obtained in ExaBayes from Ref_SNP-based dataset. (D) Species tree obtained in SVDquartets from Ref_SNP-baseddatase.

**Supplementary Figure S5.** The plot of pseudolikelihood score.

## Supplementary Tables

**Supplementary Table S1.** Sampling collection information in this study.

**Supplementary Table S2.** Qualitative (QL) and quantitative (QN) characters included in the morphometric analyses.

**Supplementary Table S3.** Qualitative (QL) and quantitative (QN) data of vegetative characters in the morphometric analyses.

**Supplementary Table S4.** Qualitative (QL) and quantitative (QN) data of reproductive characters in the morphometric analyses.

**Supplementary Table S5.** Regression coefficients from the discriminant function analysis of vegetative traits.

**Supplementary Table S6.** Regression coefficients from the discriminant function analysis of reproductive traits.

**Supplementary Table S7.** Mean value of vegetative traits for analyses of phylomorphospace.

**Supplementary Table S8.** Regression coefficients from the principal component analysis of vegetative traits.

**Supplementary Table S9.** Mean value of reproductive traits for analyses of phylomorphospace.

**Supplementary Table S10.** Regression coefficients from the principal component analysis of reproductive traits.

**Supplementary Table S11.** Tree statistics.
